# A spatial case-control study on symptomatic and inapparent primary dengue infections in an endemic city in Brazil

**DOI:** 10.1590/S1678-9946202466012

**Published:** 2024-02-19

**Authors:** Gerusa Figueiredo, Francisco Chiaravalloti, Sérgio Campos, Alessandra Cristina Guedes Pellini, Alvina Clara Felix, Expedito Luna

**Affiliations:** 1 Universidade de São Paulo, Faculdade de Medicina, Departamento de Medicina Preventiva, São Paulo, São Paulo, Brazil; 2 Universidade de São Paulo, Faculdade de Medicina, Instituto de Medicina Tropical de São Paulo, São Paulo, São Paulo, Brazil; 3 Universidade de São Paulo, Faculdade de Saúde Pública, Departamento de Epidemiologia, São Paulo, São Paulo, Brazil; 4 Universidade Nove de Julho, Programa de Pós-Graduação em Cidades Inteligentes e Sustentáveis, São Paulo, São Paulo, Brazil; 5 Universidade de São Paulo, Faculdade de Medicina, Instituto de Medicina Tropical de São Paulo, Laboratório de Virologia, São Paulo, São Paulo, Brazil

**Keywords:** Inapparent dengue, Dengue virus infection, Cohort study, Spacial case control study

## Abstract

We conducted a spatial case-control study nested in a dengue incidence cohort to explore the role of the spatial and socioeconomic factors in the proportion of symptomatic (cases) and inapparent primary dengue virus infections (controls). Cohort participants were children and adolescents (2 to 16 years of age) at the beginning of the follow-up. Case definitions were, for symptomatic cases, fever plus a positive lab result for acute dengue (NS1, RT-PCR, ELISA IgM/IgG), and for inapparent infection a positive result for dengue IgG (ELISA) in subjects without symptoms and with a previously negative result at baseline. The covariates included sociodemographic factors, residential location, and socioeconomic context variables of the census tracts of residence of cases and controls. We used principal component analysis to reduce the contextual covariates, with the component values assigned to each one based on their residences. The data were modeled in a Bayesian context, considering the spatial dependence. The final sample consisted of 692 children, 274 cases and 418 controls, from the first year of follow-up (2014-2015). Being male, older age, higher educational level of the head of the family and having a larger number of rooms in the household were associated with a greater chance of presenting dengue symptomatic infection at the individual level. The contextual covariates were not associated with the outcome. Inapparent dengue infection has extensive epidemiological consequences. Relying solely on notifications of symptomatic dengue infections underestimates the number of cases, preserves a silent source of the disease, potentially spreading the virus to unaffected areas.

## INTRODUCTION

Dengue is a global public health problem, with estimates of 390 million dengue infections per year, of which 96 million clinically manifest. It affects tropical and subtropical regions, predominantly in urban areas, where environmental conditions favor the development of the vector^1^.

Brazil is the country that reports the largest number of dengue cases in the world. In 2015 the Sao Paulo State, in Southeastern Brazil, faced its largest dengue epidemic since the disease was introduced, with more than 733,000 probable cases reported^2^. A cohort study to assess the incidence of dengue in Araraquara, a city in the Sao Paulo State, was established in August 2014. In this study, clinically detectable and inapparent cases were identified.

Inapparent dengue infections is an important component of the overall burden of dengue virus infection. These cases provide a source of infection for the mosquito, but they are not detected by health system, thus contributing to underreporting and to limitations in the prevention and control measures to be triggered. It is highly likely that inapparent dengue infections plays a role in the maintenance of dengue transmission and has extensive epidemiological consequences. Relying on notifications of symptomatic dengue underestimates cases, and further delays control efforts in areas at risk for dengue virus transmission^3^.

Globally, the mean proportion of inapparent/total dengue virus (DENV) infections, obtained in a systematic review and meta-analysis, was 80% (95% CI: 72–88), ranging from 19% (95% CI: 17–21) in the Eastern Mediterranean region to 93% (95% CI: 89-98) in the Southeast Asia region. In this publication, there were 8 studies from the regions of the Americas, with 5,363 cases and 80% (95% CI: 71-89) of inapparent infections^4^.

Prospective cohort studies have often shown higher rates of inapparent versus symptomatic infections, with inapparent to symptomatic (I:S) ratios varying by geographic area, epidemiologic context, immunological status of patients, and types of circulating DENV. The factors found in Puerto Rico were the incidence of dengue infection in a given year and the incidence of infection in the previous year, pointing to an important aspect of virus-host interactions at a population or individual level^5^. That study showed that inapparent infections were more likely to be primary infections. The same pattern was observed in Fortaleza, Brazil^6^. The Puerto Rico cohort found that children and adolescents with pre-existing immunity against DENV were at a higher risk for subsequent DENV infection during the study^5^.

In Managua, Nicaragua, it was shown that if a second DENV infection happens within a period of 2 years after the first infection, the second infection is more likely to be inapparent. However, if the time interval between first and second DENV infections is longer, this protection wanes and the infection is likely to be symptomatic^7^. In four longitudinal cohort studies, the rate of inapparent DENV infections was positively correlated with the incidence of disease the previous year, strongly supporting an important role for short-term heterotypic immunity in determining the outcome of infection. Primary and secondary infections were equally likely to be inapparent, although inapparent infections were more likely to occur in primary than in secondary DENV infections in a study in Kamphaeng Phet Province, Thailand^7,8^.

The spatial pattern of dengue cases is the result of complex interactions between the virus, the host and the vector, which can be affected by environmental conditions and is widely studied in dengue. A topic that has not been adequately explored is the proportion of symptomatic and inapparent dengue infections in this spatial dimension, seeking to explore all infection determination, in order to subsidize more effective control measures. The aim of the present study was to estimate the proportion of symptomatic and inapparent primary dengue infections and their spatial and socio demographic distribution in a case control study nested in a cohort study.

## MATERIALS AND METHODS

### Study design and population

A case-control study with data from a cohort of dengue incidence was carried out^9^. This cohort was set up with the aim of determining the incidence of dengue among children and adolescents, from 2 to 16 years of age, living in the urban area of Araraquara, a mid-size city in the Sao Paulo State, (population 212,617 in 2012), which had a GINI index of 0.50^10^. Araraquara’s urban area is divided in 308 census tracts (CTs)^11^. A cluster randomized selection was carried out considering the urban CTs of Araraquara. Seventy nine CTs were randomly selected, and within each selected CT, all households were visited to verify the presence of children in the age group of interest. After that, a pre-determined number of participants in each age stratum was randomly selected. These processes occurred between September 2014 and March 2015. A detailed description of the cohort’s methods has been published elsewhere^9^. Briefly, baseline serologic status for dengue was verified by ELISA IgG (Dengue ELISA IgG, FOCUS Technologies, Cypress, CA, USA). Active surveillance of fever was put in place since one week after the beginning of the follow up, with weekly contacts, and diagnostic tests for dengue of febrile cases: ELISA IgG and IgM (DengueVirus IgM Capture DxSelectTM and Dengue ELISA IgG, FOCUS Technologies, Cypress, CA, USA); NS1 (Platelia DENV-NS1 Ag, Bio-Rad, Marnes-la- Coquette, France); RT-PCR (in house, following CDC protocol^12^). A cross sectional serologic screening of the whole cohort was undertaken at the end of each year of follow up. The recruitment occurred from September 2014 until March 2015, and the follow up lasted 4 years.

This case-control study corresponds to the period from 2014 to 2015, period of the highest dengue incidence ever recorded in the municipality. Just participants that completed the first year of follow-up were included in the present study.

### Definitions and outcomes

#### Dependent variable

- Symptomatic and inapparent primary dengue infection
- Case definitions: symptomatic cases (at least fever, with an axillary temperature > 37.5 ^o^C) plus a positive lab result for acute dengue (NS1, RT-PCR, ELISA IgM reagent followed by IgG seroconversion and previously with a negative result in the baseline serology).
- Control definitions: inapparent dengue infection with a positive result for dengue IgG (ELISA) in the cross-sectional surveys, and with a previously negative result at the baseline serology, including those who had a symptomatic episode with negative results for dengue.

#### Independents variables

The following covariates were obtained from individual questionnaires:

- Socio-demographic factors of the individuals - sex, age, education level of the head of the household; characterization of the household (type of housing, total number of rooms and type and frequency of water supply).
- Geographic coordinates of the dengue cases and controls’ residential addresses (longitude and latitude in WGS 84). Addresses were standardized and Batchgeo and EasyMapMaker platforms were used to geocode the cases. Verification of the geocoding accuracy was performed through an active search via Google Maps.

#### Contextual covariates

To characterize the socioeconomic context of the CTs, we used the following covariates: average number of residents per household, average number of bathrooms, proportion of people aged 6 years or more who were literate, proportion of blacks and mixed ethnicity, proportion of female-head households, average household income and average income of female-head households. We obtained these covariates from the IBGE (Brazilian Institute of Geography and Statistics, the Brazilian official statistics agency, 2010 Census). We reduced the complexity of this group of covariates using the principal component analysis statistical technique, and considered all urban CTs. The principal components obtained were used to adjust our final model to the socioeconomic context of the CTs.

## Statistical analysis

### Principal component analysis (PCA)

Firstly, we standardized the seven contextual covariates, subtracting each one from its respective average and dividing the result by its respective standard deviation. Secondly, using the R program (version 4.1.3., R Core Team, Vienna, Austria) and the *psych* package (version 2.2.5., Revelle W, Evanston, Illiniois, USA). We obtained the principal components (PCs), used the varimax process to rotate them, selected the rotated PC with eigenvalues above the unit. Finally, we obtained the scores to calculate the PCs values for each one of the urban CTs^13^.

### Building the database

**Figure 1:**
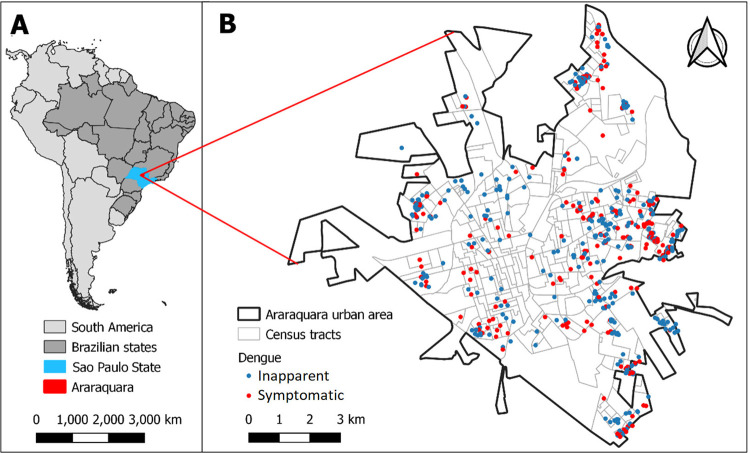
A) Municipality of Araraquara, Sao Paulo State, Brazil; B) urban area of Araraquara and the distribution of symptomatic and inapparent primary dengue infections in children and adolescentes, 2014-2015.

Each line of our database corresponded to a dengue case or control that had its address geocoded (Figure 1). We inserted in this database the individual sociodemographic covariates and the geographical coordinates. We read this database in QGIS program (Version 3.16.4, QGIS Development Team, Gossau, Zürich,Switzerland), transformed it in georreference file – a shapefile. After, we changed its coordinate reference system to SIRGAS 2000 / UTM zone 22S, a projected coordinate system, allowing us to obtain the coordinates in meters. Finally, we used a geographic tool of QGIS and the CT map to attribute to each one of the dengue cases and controls the PCs values, based on their CTs of residence.

### Exploratory data analysis

We used the R Program to evaluate possible outliers, the relationship covariates with our outcome, and collinearity among the covariates, which was evaluated using the variance inflation factor (VIF), considering the existence of collinearity if VIF > 3.0^14^.

### Bayesian statistical analysis

We modeled our outcome using a Bernoulli probability distribution. We considered a latent stationary Gaussian field, based on the projected coordinates of the cases and controls, to model the spatial dependency presented in the analyzed phenomenon. We represented this Gaussian field as a Gaussian Markov random field modeled by a Matérn function^15^. We modeled our data in a Bayesian context, using the integrated nested Laplace approximations (INLA) and the stochastic partial differential equations^16^.

We began modeling only considering our outcome and the random effects. After, we introduced in the model our individual and contextual covariates (PCs). The continuous covariates were standardized by subtracting their averages and dividing by their standard deviations. We evaluated the fitness of our models using the deviance information criterion (DIC), since the model with the lowest DIC value would be the best adjusted one^17^. The posterior means of the fixed effects were presented, after exponentiation, as odds ratios (OR) with their 95% credible intervals (95% IC). For the fixed effects and random effects, we considered, respectively, non-informative and penalized complexity priors^18^.

## Ethics

The original cohort study was approved by the research ethics review board of Hospital das Clinicas of the Medical School of the University of Sao Paulo, and is registered at the National Research Ethical Evaluation System, approval Nº CAAE25706913.6.1001.0065.

## RESULTS

We obtained a sample composed of 692 children, 274 (39.6%) of them symptomatic dengue cases and 418 (60.4%) inapparent primary dengue infections. Figure 1 shows the location of the study site, and within the city the location of cases and controls. We did not include 26 cases with secondary infections from the cohort in this study. The majority of participants were below 10 years of age. There was a slightly higher proportion of boys, in comparison with the whole cohort^9^. The majority of the heads of households had an elementary or lower educational level. Most of them lived in houses, linked to the public water system, with 5 rooms, and a median of 4 dwellers.

Regarding the assays used to confirm the diagnosis of the symptomatic dengue cases, 86.5% (n=237) were confirmed by RT-PCR; 12% by NS1 (n=33); and 1.5% (n=4) by IgM detection followed by IgG seroconversion. All RT-PCR confirmed cases were due to DENV-1. As for the symptoms, referred to by symptomatic cases, the most frequent were fever (100%), headache (84.4%), drowsiness (77.9%) and loss of appetite (69.8%).

We began analyzing the contextual covariates, and the PCA produced two components with eigenvalues greater than one. The first PC (PC1) presented an eigenvalue of 4.13, with 59% of the total variability. The second (PC2) presented an eigenvalue of 1.45 and 21% of variability. Table 1 presents the correlation of the PCs and the original contextual covariates and their respective scores. PC1 is inversely correlated with average number of residents per household and proportion of blacks and mixed ethnicity, and positively correlated with the average number of bathrooms, proportion of people aged 6 years or more literate, average household income and average income of female-head households. The higher the PC1 value, the higher the socioeconomic level of a CT, since it corresponds to areas with lower number of residents per household, lower proportion of blacks and mixed ethnicity, higher proportion of literate people and higher income of those responsible. The proportion of female-head households showed a low correlation with the PC1, not being represented by this component. PC2 stands out for its positive association with the proportion of female-head households, representing a different socioeconomic aspect of the CTs from those represented by PC1. The higher the PC2 value, the higher the proportion of female-head households of a CT.

**Table 1:** Correlation coefficients between the principal components with the original contextual covariates and their respective scores, urban area of Araraquara, Sao Paulo State, 2010*.

Contextual covariates	PC1**		PC2**	
	
	CC***	Score	CC***	Score
Average number of dwellers per household	-0.61	-0.07	-0.61	-0.38
Average number of bathrooms	0.90	0.25	-0.05	-0.18
Proportion of people aged 6 years or more literate	0.71	0.13	0.41	0.21
Proportion of blacks and mixed ethnicity	-0.84	-0.18	-0.34	-0.14
Proportion of female-head households	-0.10	-0.16	0.89	0.70
Average household income	0.95	0.27	-0.05	-0.18
Average income of female-head households	0.91	0.24	0.05	-0.10

*contextual covariates obtained from 2010 Census; **principal component; ***correlation coefficient.

We did not find outliers in the individual and contextual covariates, and their frequencies and relationships with the response variable are presented in Table 2. We did not find collinearity among the covariates, since the VIF values were all smaller than 1.19.

**Table 2:** Individual and contextual covariate frequencies considering the child and adolescent symptomatic and inapparent dengue cases, urban area of Araraquara, Sao Paulo State, Brazil, 2014 to 2015.

Covariate*	Category	Assintom (418)		Sintom (274)		Total (692); (100%)	
		
		n	%**	n	%**	n	%***
Sex	Fem	214	65.0	115	34.9	329	47.5
	Masc	204	56.2	159	43.8	363	52.5
Age group	2 a 5	121	70.3	51	29.7	172	24.9
	6 a 9	130	59.9	87	40.1	217	31.4
	10 a 13	106	57,3	79	42.7	185	26.7
	14 a 17	61	51.7	57	48.3	1118	17.1
Attends school	Não 0	23	76.7	7	23.3	30	4.3
	Sim 1	395	59.7	267	40.3	662	95.7
Schooling of the head of household	Elementary or lower	213	65.9	110	34.1	323	46.7
	High school	170	56.1	133	43.9	303	43.8
	Higher education	35	53.0	31	47.0	66	9.5
Type of household	Apartment	10	83.3	2	16.7	12	1.7
	House	408	60	272	40.0	680	98.3
Number of dwellers*	2 or 3	82	65.1	44	34.9	126	18.2
	4	136	58.9	95	41.1	231	33.5
	5	89	55.6	71	44.4	160	23.1
	6 to 12	111	63.4	64	36.6	175	25.2
Number of rooms	2 to 4	78	75.0	26	25.0	104	15.0
	5	181	57.6	133	42.4	314	45.4
	6	70	58.8	49	41.2	119	17.2
	7 to 14	89	57.4	66	42.6	155	22.4
Water supply	Piped in the property	23	69.7	10	30.3	33	4.8
	Piped in at least one room	395	59.9	264	40.1	659	95.2
Water shortage frequency	Once a week or month	36	62.1	22	37.9	58	8.4
	Rarely/never	382	60.3	252	39.7	634	91.6
PC1* (in quartiles)	-1.9612 to -1.1550	109	63.0	64	37.0	173	25
	-1.1551 to -0.8285	103	55.7	82	44.3	185	26.7
	-0.8286 to -0.3842	101	65.2	54	34.8	155	22.4
	-0.3843 to 3.7837	105	58.7	74	41.3	179	25.9
PC2* (in quartiles)	-2.5977 to -1.0295	98	63.2	57	36.8	155	22.5
	-1.0296 to -0.4146	107	56.3	83	43.7	190	27.5
	-0.4147 to 0.4082	110	63.2	64	36.8	174	25.1
	0.4083 to 2.5593	103	59.5	70	40.5	173	25

*numeric covariates that were categorized; **calculated by line;***calculated by column.

We obtained a DIC of 916.5, considering the intercept and random effect model. When we included the individual and contextual covariates, this value reduced to 892.9, showing a better adjustment. Table 3 shows its results, where we see the effects of the individual covariates on our outcome, adjusted by the contextual covariates, and taking into account the spatial autocorrelation of the study phenomenon. The contextual covariates did not show association with the outcome. From the individual covariates, the following ones were associated with the outcome: sex – male, with an increase of 56% in the odds in relation to female; age – the increase of one standard deviation in the age is associated with an increase of 34% in the odds; education of the household head – median and higher education are associated, respectively, with an increase of 75% and 108% in the odds in relation to elementary or lower education. Notably, the 95% IC of covariate total number of rooms is in the limit of the significance and could be considered a risk factor for dengue symptoms (an increase of one standard deviation is associated with an increase of 17% in the odds). Another point to be highlighted, even considering the non-significant result, is that living in a house, instead of an apartment, increases the odds by 453%.

**Table 3:** Posterior means and 95% credible intervals (95% CI) presented as odds ratios (OR)* for the individual and contextual covariates in the final model for dengue cases and controls, urban area of Araraquara, Sao Paulo State, 2014 to 2015.

Covariates	Categories	OR	CI 95%	DIC
Intercept		0.05	0.00 to 0.45	892.9
PC1		0.98	0.81 to 1.19	
PC2		1.02	0.84 to 1.24	
Sex	Female	1	1.09 to 2.19	
	Male	1.56		
Age		1.34	1.12 to 1.61	
Attends school	No	1	0.64 to 4.44	
	Yes	1.84		
Schooling of the head of household	Elementary or less	1	1.16 to 2.25	
	High school	1.75		
	Higher education	2.08	1.04 to 3.74	
Type of household	Apartment	1	0.83 to 22.18	
	House	5.53		
Number of dwellers		1.00	082 to 1.21	
Number of rooms		1.17	0.97 to 1.41	
Water supply	Piped in the property	1	0.59 to 3.15	
	Piped in just one room	1.47		
Water shortage frequency	Once a week or month	1	0.70 to 2.74	
	Rarely/never	1.45		

^*^Bayesian statistical analysis.

## DISCUSSION

There is a scarcity of studies in the literature that comparatively analyze symptomatic dengue cases and inapparent dengue infections, especially considering context variables of where the individuals live. In this study, we set out to analyze the demographic and socioeconomic differences, at an individual and contextual level, of symptomatic dengue cases and inapparent dengue virus infections in children and adolescents from 2 to 16 years of age.

Factors pointed out in some studies comparing symptomatic and inapparent rates relate to the incidence of dengue in a given year and in the previous one, the virus serotype and the number of circulating serotypes, which may influence the clinical variations of dengue in terms of the percentage of these presentations^5,6^. This is of practical importance as inapparent infections are not detected in routine surveillance and can only be captured in the context of prospective cohort or index cluster studies.

As reported by Li *et al*.^4^, the mean proportion of inapparent/total DENV infections was 80% globally and in the American region, the same proportion was found - 80% (95% CI: 71-89). Prospective cohort studies have often shown higher rates of inapparent versus symptomatic infections, with inapparent to symptomatic (I:S) ratios varying by geographic area and epidemiologic pattern. A secondary infection that occurred within a 2-year period after the primary infection was more likely to be inapparent in Managua, Nicaragua, and in Fortaleza, Brazil^6,8^. The opposite happened in Kamphaeng Phet Province, Thailand where inapparent infections were more likely to occur in primary than in secondary DENV infections^19^. In our study we decided to exclude the cohort participants who had evidence of previous DENV infections.

In the present study, the following variables were associated with a greater chance of presenting dengue symptoms at the individual level: being male; older age; higher educational level of the head of the family and a larger number of rooms in the household. Although not statistically significant, living in a house compared to an apartment conferred an increase in the odds ratio of being symptomatic, with a magnitude of 3.51. The contextual covariates, analyzed using the two main components (PC1 and PC2), did not present, per se, association with the outcome, but remained in the final model due to the adjustment on the other variables. It is noteworthy that the municipality of Araraquara has a median GINI index (0.50), indicating a population without major socioeconomic disparities.

In the literature there is a large gap in comparing socioeconomic factors of symptomatic cases and inapparent infections. So our analysis on these topics will be limited to making a parallel with symptomatic case studies. In a cohort study of schoolchildren aged 3 to 13 years in Fortaleza (CE), Brazil, no differences were found in prevalence of antibodies according to sex, age group and education, but in relation to income, the higher the purchasing power, the greater the prevalence of antibodies, which suggested greater transmission of the infection in the economically more favored classes. Other authors, who studied the spatial pattern of dengue incidence distribution and its relationship with the income variable in the city of Manaus, Amazonas State, Brazil, between 2000 and 2010, did not find an association between per capita income and the incidence of dengue in the city’s neighborhoods^20^. In our study, symptomatic dengue was associated with higher education of the head of the family and a larger number of rooms in the household, socioeconomic indicators, which can be interpreted as a proxy for greater access to information and health care for these families, which may be more attentive to the symptoms and signs presented by their children.

In the cohort in which this case-control study was nested, the distribution according to sex was almost even, with a slight predominance of girls. In a pediatric cohort in Managua, Nicaragua, overall inapparent and symptomatic dengue infection were equally distributed by sex. In the present study, we found a greater chance of presenting symptoms among male children and adolescents.

As for age, it is known that younger children present with milder symptoms or even inapparent infections, although sudden severe cases occur. Our study showed a statistically significant association of symptomatic cases with older ages. In Managua, Nicaragua, the mean age of infection was 7.1 years of age for symptomatic dengue cases and 5.9 for inapparent dengue virus infection, a 1.2 year difference.

In the literature, it is described that in children, when symptomatic, the clinical picture of dengue presents itself as an acute febrile syndrome, with nonspecific signs and symptoms, such as asthenia, drowsiness, refusal of food and fluids, vomiting, diarrhea or loose stools. In children under five years of age, the onset of the disease may go unnoticed, and the first manifestation may be a severe condition^21,22^. They represent a special group, as they have a higher risk of developing severe forms of the disease^23^. The clinical presentation of the symptomatic cases in this study consisted of unspecific symptoms, being the more frequent headache, somnolence and loss of appetite^9^. This may be due to the exclusion of participants had a primary infection, once they have a greater chance of presenting symptoms at the individual level when experiencing a secondary. dengue virus infection. There were no severe cases.

The Fortaleza study also addressed the frequency of symptomatic cases and inapparent infections. In the 1^st^ year of follow-up, 32.8% of the participants seroconverted, 20.3% with inapparent infections. The authors highlighted the epidemiological importance of inapparent primary infections, since these cases represent a silent source of the disease. As there is no clinical manifestation, infected individuals do not seek medical care and are therefore are not notified in the epidemiological surveillance system^24^. In the present study, the number of inapparent infections surpassed the number of symptomatic ones with 419 and 301 respectively, once again, the importance of inapparent infections.

The present study, in addition to being unprecedented, uses several analytical techniques that enable a robust analysis. Principal component analysis (PC) reduced seven variables from the 2010 Population Census into just two components representing the socioeconomic level of the studied region, which explained 80% of the total variability of the original covariates. In addition, the geocoding of the addresses of residence of cases and controls allowed the attribution of contextual variables to each individual, depending on the CT of residence. Finally, the Bayesian statistical analysis enabled the modeling of the dependence of the phenomenon, together with the individual and contextual covariates of the study subjects.

Although the contextual covariates, analyzed using the two main components (PC1 and PC2), showed no association with the outcome, municipalities with a high GINI index, such as the municipality of Sao Paulo (0,645)^10^, could expose more inequalities and accentuate the importance of context variables.

A limitation of the study is the low number of individuals who lived in an apartment (1.8% of the total), which may have limited the statistical power of this analysis. Another limitation concerns the fact that we decided to exclude the cohort participants with evidence of previous dengue virus infection infection which made it impossible to analyze the secondary and post-secondary responses.

## CONCLUSION

Inapparent dengue virus infection has extensive epidemiological consequences. Relying solely on notifications of symptomatic dengue underestimates the number of cases, preserves a silent source of the disease, potentially spreading the virus to unaffected areas, however infected by the vector, further delaying control efforts in areas at risk for virus transmission from dengue. Thus, inapparent infections need to be considered, especially for their impact on the dynamics of disease transmission and the effectiveness of prevention and control measures. In the present analysis no contextual variables were associated to being a symptomatic or inapparent primary dengue virus infection. However, the association in the individual level with the educational level of the head of the household (the higher the educational level, the higher proportion of symptomatic cases) variable that in Brazil is a proxy of socioeconomic status, suggests that these factors may play a role in determining the frequency of symptomatic or inapparent primary episodes of dengue virus infection.
